# TRIP-1 via AKT modulation drives lung fibroblast/myofibroblast trans-differentiation

**DOI:** 10.1186/1465-9921-15-19

**Published:** 2014-02-15

**Authors:** Michael F Nyp, Angels Navarro, Mohammad H Rezaiekhaligh, Ricardo E Perez, Sherry M Mabry, Ikechukwu I Ekekezie

**Affiliations:** 1 Department of Pediatrics, Section of Neonatal-Perinatal Medicine, Children’s Mercy Hospitals & Clinics, 2401 Gillham Road, 64108 Kansas City, MO, USA; 2 Department of Pediatrics, University of Missouri-Kansas City School of Medicine, Kansas City, MO, USA

**Keywords:** Type II TGFβ receptor interacting protein 1 (TRIP-1), Eukaryotic translation initiation factor-3 (eIF3), Pulmonary fibroblasts, α-smooth muscle actin (α-SMA), Fibrosis

## Abstract

**Background:**

Myofibroblasts are the critical effector cells in the pathogenesis of pulmonary fibrosis which carries a high degree of morbidity and mortality. We have previously identified Type II TGFβ receptor interacting protein 1 (TRIP-1), through proteomic analysis, as a key regulator of collagen contraction in primary human lung fibroblasts—a functional characteristic of myofibroblasts, and the last, but critical step in the process of fibrosis. However, whether or not TRIP-1 modulates fibroblast trans-differentiation to myofibroblasts is not known.

**Methods:**

TRIP-1 expression was altered in primary human lung fibroblasts by siRNA and plasmid transfection. Transfected fibroblasts were then analyzed for myofibroblast features and function such as α-SMA expression, collagen contraction ability, and resistance to apoptosis.

**Results:**

The down-regulation of TRIP-1 expression in primary human lung fibroblasts induces α-SMA expression and enhances resistance to apoptosis and collagen contraction ability. In contrast, TRIP-1 over-expression inhibits α-SMA expression. Remarkably, the effects of the loss of TRIP-1 are not abrogated by blockage of TGFβ ligand activation of the Smad3 pathway or by Smad3 knockdown. Rather, a TRIP-1 mediated enhancement of AKT phosphorylation is the implicated pathway. In TRIP-1 knockdown fibroblasts, AKT inhibition prevents α-SMA induction, and transfection with a constitutively active AKT construct drives collagen contraction and decreases apoptosis.

**Conclusions:**

TRIP-1 regulates fibroblast acquisition of phenotype and function associated with myofibroblasts. The importance of this finding is it suggests TRIP-1 expression could be a potential target in therapeutic strategy aimed against pathological fibrosis.

## Background

Fibrosis, a pathobiological process resulting in tissue remodeling from overabundant extracellular matrix deposition, is a major cause of morbidity and mortality. Myofibroblasts are considered chief perpetrators of this pathology. Myofibroblasts are a trans-differentiated fibroblast phenotype, identified by alpha smooth muscle actin (α-SMA) expression [1,2]. They are avid extracellular matrix synthesizers and have enhanced collagen contraction ability. Myofibroblasts are more resistant to apoptotic signaling/induction than fibroblasts. This is particularly important because a failure of proper apoptotic termination, a consequence of an inability of epithelial cells to successfully restore damaged epithelium, is a significant contributor to pathological fibrosis. Regulation of fibroblast acquisition of this trans-differentiated phenotype is a complex process modulated at multiple levels: transcriptional, translational, and post-translational. Factors driving this trans-differentiation include transforming growth factor β1 (TGFβ1) ligand which has also been implicated as a central mediator of fibrosis and has been shown to induce α-SMA expression in TGFβ1-treated fibroblasts [3-14].

Type II TGFβ receptor interacting protein-1 (TRIP-1) is a WD-40-containing endogenous protein. It was initially identified as a phosphorylation target of the TGFβ type II receptor kinase capable of modulating TGFβ1 response, but later as a functional component of eukaryotic translation initiation factor-3 (eIF3) multi-protein complexes [15-17]. However, unlike most other eIF3 component proteins, it was shown to also exist independently in the cytoplasm, free from eIF3 complex, suggesting TRIP-1 may interact with other factors and have functions not related to protein translation [15,18,19]. Indeed, Shue et al. demonstrated that TRIP-1 can bind tartrate-resistant acid phosphatase (TRAP) in osteoblasts causing cytodifferentiation to osteoclasts [20]. More recently they showed that TRIP-1 is secreted in the provisional bone matrix and is not only present in the cytosol, but also in the nucleus of osteoblasts [21]. We have reported that TRIP-1 is a negative regulator of fibroblast collagen contraction and modulates epithelial-mesenchymal transition (EMT) of lung epithelial cells, which would suggest it has the potential to influence execution of fibrotic pathology [22-24]. Myofibroblasts are the critical effector cells in the pathogenesis of fibrotic remodeling, but whether or not TRIP-1 modulates fibroblast trans-differentiation to a myofibroblast phenotype has not been explored.

In the current work, we report that down-regulation of TRIP-1 expression in primary human lung fibroblasts has the promotive effect of inducing trans-differentiation to the myofibroblast phenotype, while over-expression has inhibitory effects. Remarkably, the effects of down-regulating TRIP-1 were not abrogated by blockage of TGFβ1 ligand Smad3 activation, or by knocking-down Smad3 expression. Moreover, loss of TRIP-1 enhanced the ability to resist apoptosis and collagen contraction ability in the cells. This effect we uncovered is due to a TRIP-1 dependent rise in the phosphorylated-AKT levels that promotes accumulation of α-SMA and prolongs cell survival in a collagen matrix. These findings are important because they reveal TRIP-1 in a novel regulatory role of modulating fibroblast acquisition of phenotype and function associated with myofibroblasts, which may have implications for development of potential therapies for pathological fibrosis.

## Methods

Study was performed using commercially available human fibroblast cell lines. No animal or human subjects were enrolled or used in this study thus IRB or IACUC approvals were not applicable.

### Cells, reagents

Human Lung Fibroblasts of fetal (HLF-F) or adult (HLF-A) origin were purchased from Cell Applications, Inc. (San Diego, CA) and grown in HLF growth media (used passages 3–5). SB-431542 was from Sigma-Aldrich (St. Louis, MO), Akt inhibitor II from Calbiochem (EMD Millipore, Billerica, MA) and Rhodamine-Phalloidin was from Cystoskeleton, Inc. (Denver, CO).

### siRNA-mediated gene silencing

TRIP-1 (siRNA ID no. 13735), control (siRNA-1 AM4611) and Smad3 (siRNA ID no. s8400) were from Ambion (Applied Biosystems, Austin, TX). HLF-F were transfected with Lipofectamine RNAiMAX (Life Technologies, Carlsbad, CA), according to the manufacturer’s protocol, to final concentration 50 nM of control or TRIP-1 (EIF3I) siRNA. For some experiments Smad3 siRNA was added (5 nM). Lysates or set-up of collagen contraction assays was performed 2 days after transfection.

### Plasmid-mediated AKT/TRIP-1 over-expression

An active-form AKT plasmid (pSG5-AKT) was a gift from Dr A. C. Larner (developed by Burgering and Coffer [25]). pcDNA4/hTRIP-1-V5/His and pcDNA4/LacZ were generated using pcDNA4/TO/myc-His vector from Life Technologies (Carlsbad, CA). For AKT, 70% confluent HLF-F were transfected with Lipofectamine and Plus reagent according to instructions, trypsinized 24 hours later and embedded in collagen for contraction or apoptosis analysis. For TRIP-1, HLF-A cells were starved in 0.5% FBS-containing media, treated with 5 ng/ml TGF-1 in 0.5% FBS-media for 24 hours and transfected with jetPRIME (Polyplus-Transfection Inc., New York, NY) according to manufacturer’s instructions. Cells were plated onto glass coverslips 24 hours later, and stained 24 hours later.

### TGFβ receptor and Akt inhibitor

TRIP-1 siRNA-transfected HLF-F were treated with vehicle (DMSO) or with 10 μM solution of TGFβ receptor inhibitor SB431542 in DMSO for 24 hours. This concentration of SB431542 has been shown to block TGFβ1 ligand signaling [24,26]. For Akt inhibition, Akt inhibitor II at 40 μM was added immediately after transfection and left 48 hours. This concentration has been shown to inhibit Akt activity in cells [27].

### Collagen contraction

Type I collagen gels were prepared by mixing cold rat tail HC collagen (BD Biosciences) with 10X DMEM (Sigma-Aldrich, St Louis, MO) on ice, neutralizing with 1 M NaOH and diluting to 1 mg/ml collagen. Fibroblasts were trypsinized, resuspended in cold, serum-free DMEM, and added to the collagen mixture at a final concentration of 5 × 10^5^ cells/ml with 0.75 mg/ml as final collagen concentration [22]. Aliquots (in triplicate) were cast onto 24-well plates and allowed to solidify at 37°C (30–45 minutes), after which serum-free media was added, incubated, periodically observed for contraction, and photographed. Experiments were performed a minimum of three times.

### Western blot

Total cell lysates were prepared (unless specified in figure legend), and proteins were separated by SDS-PAGE, transferred to PVDF membranes and detected with ECL from GE Healthcare Biosciences (Piscataway, NJ) as previously described [22], using polyclonal rabbit anti-TRIP-1 from Abcam (1:1000), mouse monoclonal antibodies against smooth muscle actin (1:1000) and tubulin (1:10000) from Sigma-Aldrich and P-Ser473-Akt (1:1000), Smad3 (rabbit monoclonal C67H9, 1:1000), Cleaved Caspase-3 (rabbit monoclonal D175, clone 5A1E, 1:1000 using nitrocellulose 0.2 μm pore size instead of PVDF membranes) from Cell Signaling Technologies, and anti-actin (polyclonal goat I-19, 1:1000) from Santa Cruz Biotechnologies (Santa Cruz, CA). Western-blots were developed using ECL (chemiluminescence), and densitometric scans were quantified using Alpha-Innotech and AlphaEaseFC Imaging Software (Protein Simple, Santa Clara, CA).

### Immunofluorescence

Coverslips were rinsed with warm PBS, then cells were fixed using 3.7% paraformaldehyde in PBS for 10 minutes at room temperature, rinsed twice with PBS, permeabilized with 0.1% Triton X-100 in PBS for 5 minutes and rinsed twice with PBS again. Cells were then stained with Rhodamine-Phalloidin from Cytoskeleton, Inc (Denver, CO) according to manufacturer’s recommendations (3–5 units/coverslip, in PBS containing 0.5% BSA, for 30 minutes at room temperature). After rinsing twice with PBS, staining for V5-TRIP-1 was performed after blocking with 3% BSA in PBS, using a mouse monoclonal antibody against V5 (Life Technologies, (Carlsbad, CA)) at a 1/500 dilution, after which cells were rinsed, incubated with a secondary anti-mouse AlexaFluor-488 from Life Technologies (Carlsbad, CA), and then coverslips were mounted onto slides using DAPI-containing Vectashield (Vector Laboratories, Burlingame, CA) after three washes in PBS. For α-SMA staining, a rabbit antibody from Abcam Ltd (Cambridge, MA) was used at 1/500 dilution in conjunction with the V5 antibody, and a secondary anti-rabbit Alexa Fluor 564. Pictures were taken using a CCD camera coupled to an Olympus BX60 fluorescence microscope.

### TUNEL assay

Twenty-four hours following siRNA transfection, fibroblasts were embedded into collagen gels and, at the appropriate times, fibroblasts were analyzed for apoptosis using an in situ cell death detection kit (fluorescence TUNEL assay from Roche Applied Science, Indianapolis, IN) according to manufacturer’s instructions. Briefly, gels were collected, treated at 60°C for 10 minutes, then samples were spun down 3 minutes at 400×g to collect cells, washed with 10% FBS-containing media, then plated onto glass slides using a cytospin; recovered cells were fixed with 4% paraformaldehyde for 1 hour at room temperature and permeabilized with 0.1% Triton X-100 for 2 minutes at 4°C. TUNEL reaction mixture was added to the cells, incubated for 60 minutes at 37°C in the dark, washed twice with PBS, then mounted with coverslips using Vectashield with DAPI, and analyzed by fluorescence microscopy.

### Statistical analysis

Results are expressed as means ± SE of data obtained. Statistical analysis was performed with student’s t-test for paired comparisons. A value of *P* < 0.05 was considered significant.

## Results

### Loss of TRIP-1 in human lung fibroblasts induces alpha-smooth muscle actin expression promoting myofibroblast phenotype and function

**Figure 1:**
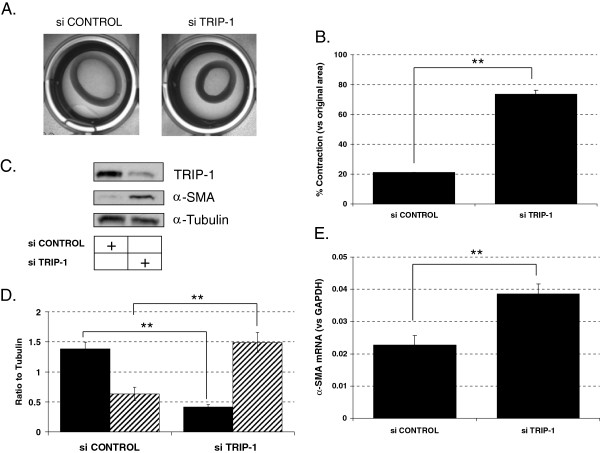
**Loss of TRIP-1 in human lung fetal fibroblasts enhances collagen contraction ability and induces expression of alpha-smooth muscle actin (**α**-SMA). A**: Collagen contraction assays using HLF-F transfected with control siRNA (left) or TRIP-1 siRNA (right) at 7 days; **B**: % area contracted respect to original area is shown based on analysis of images like the ones shown in **A)** with means ± SE shown (**P < 0.05, *n* = 6); **C**: analysis of α-SMA expression in HLF-F cells transfected with control or TRIP-1 siRNA (100 nM); cells were transfected and 48 h later lysates were made and analyzed by western blot for TRIP-1, α-SMA and α-Tubulin (as a loading control); **D**: normalized values of TRIP-1 expression (black boxes) or α-SMA expression (hatched boxes) in control siRNA (left) or TRIP-1 siRNA (right) transfected cells, with means ± SE shown (**P < 0.05, *n* = 3); **E)** analysis of α-SMA mRNA expression by real-time PCR in control siRNA or TRIP-1 siRNA. Values are normalized to GAPDH mRNA. Experiments were performed at least three times and one representative experiment is shown, with means ± SE shown (**P < 0.05, *n* = 3).

Alpha-smooth actin (α-SMA), a hallmark of myofibroblasts, plays a central role in forming stress fibers, and its level of expression is reported to correlate with the ability of (myo)fibroblasts to contract collagen [1]. We previously found that fetal human lung fibroblasts (HLF-F) lesser ability for collagen contraction is due to higher TRIP-1 protein expression [22]. To assess whether TRIP-1 drives fibroblast acquisition of myofibroblast phenotype and function in human lung fibroblasts, TRIP-1 siRNA-transfected versus control siRNA-transfected HLF-F were embedded in a 3D collagen matrix and studied for collagen contraction response and α-SMA expression. As shown in Figure 1A and Figure 1B, TRIP-1 siRNA transfection of HLF-F shows enhanced collagen contraction when compared to the control siRNA-transfected HLF-F. Furthermore, α-SMA expression was higher in the TRIP-1 siRNA-transfected fibroblasts when compared to control siRNA-transfected fibroblasts, as shown in Figure 1C and Figure 1D. In addition, TRIP-1 siRNA transfected HLF-F showed increased caldesmon and calponin expression (Additional file 1: Figure S1). Importantly, the increase in α-SMA expression was present at the mRNA level, as detected by real-time PCR analysis (Figure 1E). Because TRIP-1 is known to regulate TGFβ signaling and TGFβ signaling plays an important role in regulating α-SMA expression, we evaluated PAI-1 and CTCF gene expression to assess TGFβ pathway activation. Interestingly, PAI-1 and CTCF mRNA expression in TRIP-1-transfected fibroblasts was lower at baseline but showed a robust response when exposed to TGFβ when compared to control siRNA-transfected fibroblasts (Additional file 1: Figure S2).

### α-SMA expression mediated by loss of TRIP-1 is mostly independent of TGFβ1 signaling

**Figure 2:**
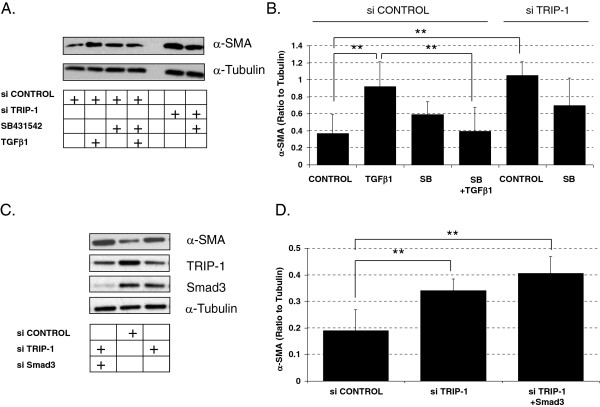
**Effect of loss of TRIP-1 on α-SMA expression is independent of TGFβ receptor signaling and Smad3. A**: HLF-F transfected with control siRNA or TRIP-1 siRNA were treated with either vehicle or with 10 μM solution of TGFβ receptor inhibitor (SB431542) for 24 h, after which whole cell lysates were prepared and samples run on SDS-PAGE and analyzed by western-blot for α-SMA protein; Western blot shows a reduction in TGFβ- induced α-SMA in controls treated with SB431542, while the α-SMA was not significantly different in the TRIP-1 siRNA treated fibroblasts when treated with SB431542. TGFβ1 treatment was used as a positive control for the inhibitor; α-Tubulin was used as a loading control; **B**: normalized values of α-SMA expression in control siRNA (left) or TRIP-1 siRNA (right) transfected cells, with means ± SE shown (**P < 0.05, *n* = 3) showing a significant difference in TGFβ-induced α-SMA in the controls when treated with SB431542 but no difference in the TRIP-1 siRNA fibroblast treated with SB431542; **C**: HLF-F cells were transfected with control siRNA, TRIP-1 siRNA or TRIP-1 and Smad3 siRNA and 48 hours later lysates were prepared and analyzed by western-blot for α-SMA expression showing increased α-SMA in TRIP-1 siRNA is independent of Smad3 activity; **D**: normalized values of α-SMA expression in control siRNA (left column), TRIP-1 siRNA (middle column) or TRIP-1 and Smad3 siRNA (right column) transfected cells, with means ± SE shown (**P < 0.05, *n* = 3). Experiments were performed three times and one representative experiment is shown.

**Figure 3:**
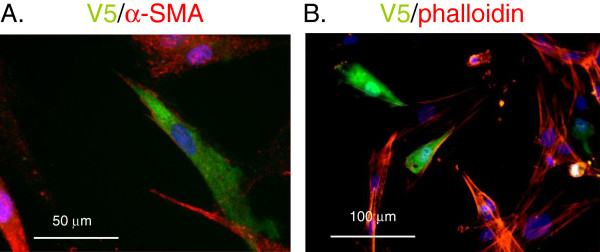
**Decreased α-SMA expression and less stress fibers in HLF-A transfected with TRIP-1 plasmid.** HLF-A cells were left untreated or treated with 5 ng/ml TGF-β1 in 0.5% FBS for 3 days, then transfected with V5-TRIP-1 plasmid and analyzed 2 days later. Panels **A** and **B** show immunofluorescent stains of V5-transfected cells, stained with V5 antibody in green and α-SMA **(panel A)** or Rhodamine-Phalloidin **(panel B)** in red. Blue corresponds to DAPI (nuclei). Experiment was performed three times and one representative experiment is shown.

Activation of TGFβ1 signaling in fibroblasts leads to increased α-SMA expression and enhanced collagen contraction. Choy et al. had previously reported on the regulatory role TRIP-1 has in TGFβ1 signaling [17]. Therefore, we assessed whether TRIP-1 effects on collagen contraction and α-SMA expression were due to its known regulatory role on TGFβ1 signaling. We blocked TGFβ1 signaling using SB431542, a TGFβ receptor 1 inhibitor, in TRIP-1 siRNA-transfected HLF-F. However, α-SMA expression remained elevated in the TRIP-1 siRNA-transfected HLF-F treated with SB431542 when compared to TRIP-1 siRNA-transfected HLF-F treated with vehicle (Figure 2A and Figure 2B). To confirm that the TRIP-1 role in α-SMA accumulation was independent of Smad3 activation, we blocked Smad3 signaling by siRNA Smad3 transfection in TRIP-1 siRNA-transfected HLF-F. Persistent α-SMA expression was observed following double transfection of these HLF-F when compared to controls (Figure 2C and Figure 2D), suggesting TRIP-1 regulates α-SMA expression through a TGFβ1 ligand independent mechanism. To further assess the role TRIP-1 has on regulating α-SMA expression and its ability to modify α-SMA expression in fibroblasts that have already been induced with TGFβ1, we treated adult HLF with TGFβ1 followed by V5-TRIP-1 transfection. As shown in Figure 3A, staining of V5-TRIP-1-transfected, TGF-β1- treated fibroblasts with V5 and α-SMA antibodies demonstrated a decreased expression of α-SMA in transfected cells compared to cells that do not express V5-TRIP-1, and a decrease of actin stress-fibers detected with Rhodamine-Phalloidin in the V5-TRIP-1-expressing cells (Figure 3A and 3B). Thus, over-expression of TRIP-1 suppresses α-SMA expression stimulated by TGFβ1 ligand in human lung fibroblasts.

### Loss of TRIP-1 leads to prolonged cell survival and resistance to apoptosis in human lung fibroblasts

**Figure 4:**
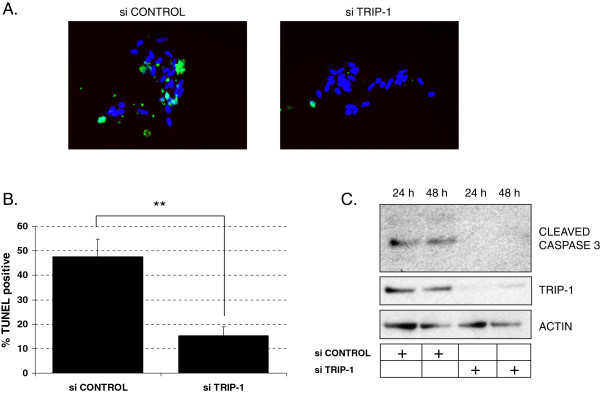
**Loss of TRIP-1 in human lung fetal fibroblasts leads to decreased apoptosis in a collagen matrix. A**: TUNEL staining in HLF-F cells transfected with control siRNA (left) or TRIP-1 siRNA (right). Cells were transfected, and 2 days later embedded in a type I collagen matrix and allowed to contract matrix for 5 days, then cells were recovered from the gel and staining performed as described in Methods. Staining in green shows positive TUNEL, while blue corresponds to DAPI; **B**: Quantification of % positive TUNEL stained cells (apoptosis) based on analysis of pictures like the ones shown in A. Experiment was repeated three times, one experiment is shown. Means ± SE shown (**P < 0.05, *n* = 3); **C**: control siRNA or TRIP-1 siRNA-transfected cells were embedded in collagen matrix, gels were collected after 24 or 48 hours and lysed directly in sample buffer (Laemli); western-blot for cleaved caspase 3 is shown. Actin is shown as a protein loading control; one representative Western-blot experiment is shown, out of three performed.

The ability of myofibroblasts to resist apoptosis signaling is linked to the development of pathological fibrosis, while the termination of fibroblast contractility response is important for nonfibrotic wound healing. To assess the role of TRIP-1 expression in fibroblast apoptosis response, we analyzed the degree of apoptosis by TUNEL assay between the TRIP-1 siRNA-transfected and control siRNA-transfected HLF-F after 5 days in the collagen gel. As seen in Figure 4A and 4B, the percentage of TUNEL-positive nuclei was significantly lower in the TRIP-1 siRNA-transfected HLF-F when compared to the control siRNA-transfected HLF-F, suggesting TRIP-1 is a negative regulator of fibroblast apoptosis. Apoptosis was also detected using western-blot in samples from cells embedded in collagen gels (Figure 4C). At 24 and 48 hours after embedding in collagen, control siRNA-transfected cells show detectable levels of cleaved caspase 3 by Western-blot, which is absent in the TRIP-1 siRNA-transfected cells.

### TRIP-1 depleted fetal human lung fibroblasts have increased phosphorylated AKT levels and loss of AKT signaling negates the regulatory role of TRIP-1 on α-SMA expression

**Figure 5:**
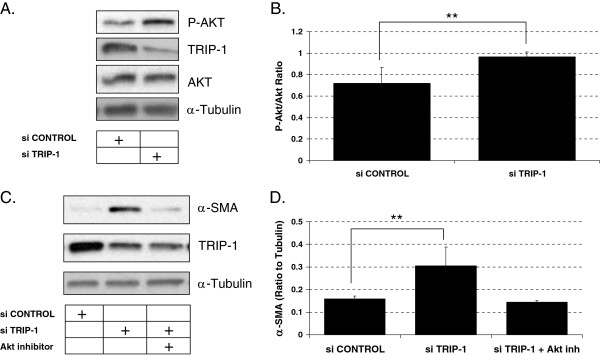
**Loss of TRIP-1 is associated with increased phosphorylation of the pro-survival protein Akt, and increased Akt activity drives α-SMA expression in cells with low TRIP-1 expression. A**: HLF-F were transfected with control siRNA or TRIP-1 siRNA and 48 hours later lysates were prepared and levels of phosphorylated Akt were analyzed by western-blot. α-Tubulin was used as a loading control; **B**: Densitometric analysis of P-Akt levels normalized to total Akt in control siRNA-transfected (left) or TRIP-1 siRNA-transfected cells (right) with means ± SE shown (**P < 0.05, *n* = 3); **C**: HLF-F were transfected with control siRNA or TRIP-1 siRNA, and treated right after transfection (or not) with 40 μM Akt inhibitor II for 2 days before lysates were prepared. Experiment was performed three times, and one representative experiment is shown, with control siRNA-transfected cells(left), TRIP-1 siRNA-transfected cells (middle) and TRIP-1 siRNA-transfected, Akt inhibitor II-treated cells (right); **D**: Densitometric analysis of α-SMA normalized to tubulin from C, including three sets of samples, with means ± SE shown (**P < 0.05, *n* = 3).

Fibroblast apoptosis signaling occurs through many pathways, but of particular interest is the interaction between integrins and ECM. Integrin-linked kinase (ILK) expression decreases in response to collagen matrix contraction leading to decreased levels of phosphorylated AKT (prosurvival form of AKT) and increased fibroblast apoptosis. We evaluated TRIP-1′s role in modulating AKT expression by measuring AKT levels in both TRIP-1 siRNA-transfected and control siRNA-transfected HLF-F. As seen in Figure 5A and 5B, the levels of phosphorylated AKT were increased in the TRIP-1 siRNA-transfected HLF-F when compared to the control siRNA-transfected HLF-F.

To further assess the role AKT plays in contractility we investigated the role AKT expression has on α-SMA expression. We attempted AKT siRNA transfection, but transfection efficiency was very poor and seemed to cause cell death (data not shown). Transfection with a plasmid construct that works as a dominant negative AKT was also toxic to the cells (not shown). We next used Akt inhibitor II, a chemical inhibitor, to block AKT signaling in TRIP-1 siRNA-transfected HLF-F. This inhibitor has been shown to block AKT activation [27], and in HLF, a 2 hour treatment with the inhibitor is able to decrease phosphorylated AKT levels as detected by Western blot (Additional file 1: Figure S3). Interestingly, α-SMA expression was abrogated in HLF-F treated with inhibitor compared to TRIP-1 siRNA transfected HLF-F (Figure 5C and D). This suggests that TRIP-1 regulates α-SMA expression through the AKT signaling pathway, and activation of AKT is required for induction of α-SMA.

### Activated AKT mediates the enhanced collagen contraction in human lung fibroblasts

**Figure 6:**
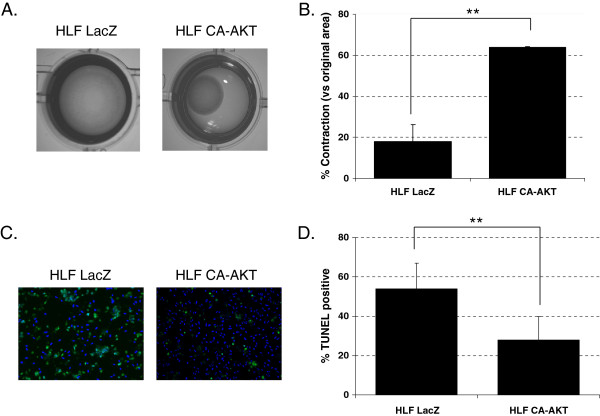
**Increased Akt activity leads to increased contractility and protection from apoptosis in collagen gels. A**: HLF-F transfected cells with control plasmid HLF LacZ (pcDNA4.0-LacZ, left panel) or an active form of Akt HLF CA-AKT (pSG5-AKT GAG, right panel) were embedded in collagen gels, and contraction was analyzed 4 days later; **B**: % area contracted in respect to original area after 4 days is shown based on analysis of images like the ones shown in **A)**; **C**: HLF-F transfected cells with control plasmid HLF LacZ (pcDNA4.0-LacZ, left panel) or an active form of Akt HLF CA-AKT (pSG5-AKT GAG, right panel) were embedded in collagen gels, and TUNEL staining was performed after 4 days in collagen matrix; **D**: % positive TUNEL cells obtained from analysis of pictures like those in **B)**. Experiments were performed three times and one representative experiment is shown for each type, with mean ± SE shown (**P < 0.05, *n* = 3).

The activation of the AKT pathway provides a survival signal for fibroblasts and has been reported to be down-regulated in fibroblasts in a collagen matrix [28]. We assessed the role the AKT pathway plays in the fibroblast contractile response by transfecting HLF-F with a control plasmid or pSG5-AKT GAG, an active form of AKT (CA-AKT). As shown in Figure 6A and 6B, the CA-AKT-transfected HLF-F demonstrated an enhanced contractile response when compared to control LacZ-transfected HLF-F. Also, the CA-AKT-transfected HLF-F underwent less apoptosis when compared to control transfected HLF-F (Figure 6C and 6D). These findings confirm the significant role that fibroblast survival through AKT activation has in sustained collagen contractile response.

## Discussion

It is generally considered that the excessive fibrogenesis and resultant extracellular matrix remodeling in pathological fibrosis is due to the presence and persistent activity of activated fibroblasts of the myofibroblast phenotype and function [29-31]. This fibroblast phenotype is not only an avid synthesizer of extracellular matrix, it also has enhanced collagen contraction ability, in addition to being more resistant to apoptosis. In the current work, we report that down-regulation of TRIP-1 expression in primary human lung fibroblasts has the promotive effect of inducing trans-differentiation to the myofibroblast phenotype and function, while over-expression has inhibitory effects. Down-regulation of TRIP-1 expression induces α-SMA expression and resistance to apoptosis, along with an increased ability for collagen contraction. Implicated mechanistically is a TRIP-1 dependent rise in phosphorylated-AKT level that leads to accumulation of α-SMA and prolongation of cell survival, and therefore, the ability to contract a collagen matrix. These findings are important because they reveal TRIP-1 in a novel regulatory role of modulating fibroblast acquisition of the phenotype and function associated with myofibroblasts—the chief perpetrator cell in fibrosis. This may have implications in development of potential therapies for pathological fibrosis.

The fibroblast’s acquisition of the myofibroblast phenotype, which is identified by accumulation of α-SMA, occurs through multiple pathways, but TGFβ1/Smad3 plays the central role [3,13,14]. Thus, excessive activation of the TGFβ1/Smad3 pathway has been linked to wound fibrosis and fibroblasts treated with TGFβ1 show induction of α-SMA [6,8,9,11]. We observed that over-expression of TRIP-1 in HLF previously treated with TGFβ1 represses α-SMA expression, while knockdown of TRIP-1 in HLF causes α-SMA induction—suggesting TRIP-1 regulates α-SMA expression. The changes were evident at both transcriptional and translational levels. However, a TRIP-1 mediated molecular pathway independent of TGFβ1 ligand must be involved because this induction of α-SMA occurred without TGFβ1 treatment and even after inhibiting TGF beta receptor 1 kinase with SB431542 and Smad3 knockdown. Interestingly, we found AKT phosphorylation was also increased in the siRNA TRIP-1 knockdown fibroblasts and that inhibiting AKT activation in these cells blocked the α-SMA induction. AKT is important to cellular activity such as cell growth and survival, and is upregulated during normal epithelial wound healing [32]. Wang et al. recently reported TRIP-1 interacts with AKT to modulate its activity level and demonstrated a direct relationship between TRIP-1 expression and AKT activity [33]. In our primary human lung fibroblasts, we found TRIP-1 depletion increased AKT activation which is in contrast to the direct relationship observed by Wang et al. in their hepatocellular carcinoma and transformed cell lines. This is not surprising given that cancer cells are notorious for a deregulation in their translation machinery. Since inhibition of TGF beta receptor 1 activation of Smad3 with SB431542 and Smad3 knockdown did not abrogate the loss of TRIP-1 effect on α-SMA induction but blocking AKT signaling did, TRIP-1 effects likely involve direct or signaling interaction with downstream factors related to the AKT signaling pathway.

Fibroblasts of the myofibroblast phenotype are generally considered to be more resistant to apoptosis and have increased collagen contraction ability. Indeed, the ability of myofibroblasts to resist apoptosis has been suggested as a central event driving the development of fibrotic disease, while appropriate termination of activated fibroblast-mediated response leads to normal fibrogenesis. We found that our human lung fibroblasts with down-regulated TRIP-1 expression were more resistant to apoptosis, consistent with the functional phenotype associated with trans-differentiation to myofibroblasts. Although apoptotic signaling in fibroblasts occurs through many pathways, it is well established that fibroblasts incorporated in type 1 collagen matrix have increased phosphorylated-AKT levels through β1 integrin dependent activation of phosphatidylnositol 3 kinase (PI3K) and integrin-linked kinase (ILK) [34]. Over time, while the fibroblasts contract the collagen matrix, this integrin pathway is down-regulated, leading to decreased levels of phosphorylated-AKT, which ultimately leads to fibroblast apoptosis. Nho et al. reported that when PTEN, a major negative regulator of the PI3K/AKT signal pathway, is not expressed in fibroblasts, phosphorylated-AKT levels are elevated and fibroblasts are resistant to collagen matrix contraction-induced apoptosis [28]. Our results demonstrate that TRIP-1 siRNA-transfected HLF-F have an increased phosphorylated-AKT signaling. Furthermore, enhanced collagen contraction and decreased apoptosis in HLF-F transfected with active AKT plasmid suggests apoptosis plays a central role in collagen contraction independently of α-SMA expression.

## Conclusion

In summary, we found that down-regulating TRIP-1 expression in primary human lung fibroblasts induces α-SMA expression, enhances resistance to apoptosis, and enhances collagen contraction ability. These changes are consistent with trans-differentiation to myofibroblast phenotype and function. In contrast, TRIP-1 over-expression inhibits α-SMA expression. Remarkably, loss of TRIP-1 effects is not abrogated by blockage of TGFβ1 ligand activation of the Smad3 pathway or by Smad3 knockdown. Rather a TRIP-1 mediated enhancement of AKT phosphorylation is implicated as the mechanism. The importance of these findings is that they reveal TRIP-1 as a novel regulator of fibroblast acquisition of phenotype and function associated with myofibroblasts and thus, a potential target in therapeutic strategy aimed against pathological fibrosis.

## Competing interests

The authors have no competing interests.

## Authors’ contributions

MFN Interpreted results of experiments, drafted manuscript, edited and revised manuscript, approved final version of manuscript. AN Performed experiments, analyzed data, interpreted results of experiments, prepared figures, edited and revised manuscript, approved final version of manuscript. MHR Performed experiments and approved final version of manuscript. REP Performed experiments and approved final version of manuscript. SMM Performed experiments, edited and revised manuscript, approved final version of manuscript. IIE Conception and design of research, analyzed data, interpreted results of experiments, edited and revised manuscript, approved final version of manuscript.

## Supplementary Material

Additional file 1 **Supplemental methods and figures.** Western-blot-Cells transfected with control siRNA or TRIP-1 siRNA were lysed 48 hours after transfection, and western-blot was performed using antibodies against caldesmon (rabbit monoclonal E89 from Abcam, 1:10,000) and calponin (rabbit monoclonal EP798Y from Abcam, 1:10,000). Real-time PCR-RNA was obtained from cells that had been transfected with control siRNA or TRIP-1 siRNA and treated with TGFβ1 5 ng/ml for 48 hours. Total RNA was isolated using Trizol, DNAse-treated, then processed for First-Strand Synthesis with SuperScript (Life Technologies) and real-time PCR was performed on BioRad MyIQ system using IQ SyberGreen-Supermix from BioRad, Inc. Experiment was performed three times and samples were run in triplicate. Figure Legends: **Figure S1.** Expression of caldesmon and calponin by western-blot is increased in cells transfected with TRIP-1 siRNA. **Figure S2.** Real-time PCR analysis of TGF-β -dependent genes A) PAI-1 and B) CTGF in cells with decreased TRIP-1 expression, with or without TGFβ 48 hours treatment. **Figure S3.** AKT inhibitor II treatment decreases phosphorylation of AKT. Cells in complete media were treated with vehicle (DMSO) or 40 μM AKT inhibitor for 2 hours, then samples were made and SDS PAGE gels run, and Western-Blot performed for P-AKT and Tubulin. Click here for file
