# Supplementary material for: TRIP-1 via AKT modulation drives lung fibroblast/myofibroblast trans-differentiation
Source: Respir Res. 2014 Feb 15;15(1):19. doi: 10.1186/1465-9921-15-19 (PMC3946032; doi:10.1186/1465-9921-15-19)
Supplement: Additional file 1 — Supplemental methods and figures. Western-blot-Cells transfected with control siRNA or TRIP-1 siRNA were lysed 48 hours after transfection, and western-blot was performed using antibodies against caldesmon (rabbit monoclonal E89 from Abcam, 1:10,000) and calponin (rabbit monoclonal EP798Y from Abcam, 1:10,000). Real-time PCR-RNA was obtained from cells that had been transfected with control siRNA or TRIP-1 siRNA and treated with TGFβ1 5 ng/ml for 48 hours. Total RNA was isolated using Trizol, DNAse-treated, then processed for First-Strand Synthesis with SuperScript (Life Technologies) and real-time PCR was performed on BioRad MyIQ system using IQ SyberGreen-Supermix from BioRad, Inc. Experiment was performed three times and samples were run in triplicate. Figure Legends: Figure S1. Expression of caldesmon and calponin by western-blot is increased in cells transfected with TRIP-1 siRNA. Figure S2. Real-time PCR analysis of TGF-β -dependent genes A) PAI-1 and B) CTGF in cells with decreased TRIP-1 expression, with or without TGFβ 48 hours treatment. Figure S3. AKT inhibitor II treatment decreases phosphorylation of AKT. Cells in complete media were treated with vehicle (DMSO) or 40 μM AKT inhibitor for 2 hours, then samples were made and SDS PAGE gels run, and Western-Blot performed for P-AKT and Tubulin. [file 1465-9921-15-19-S1.doc]

Additional file 1

SUPPLEMENTAL METHODS AND FIGURES

**Western-blot**

Cells transfected with control siRNA or TRIP-1 siRNA were lysed 48 hours after transfection, and western-blot was performed using antibodies against caldesmon (rabbit monoclonal E89 from Abcam, 1:10,000) and calponin (rabbit monoclonal EP798Y from Abcam, 1:10,000).

**Real-time PCR**

RNA was obtained from cells that had been transfected with control siRNA or TRIP-1 siRNA and treated with TGFβ1 5 ng/ml for 48 hours.

Total RNA was isolated using Trizol, DNAse-treated, then processed for First-Strand Synthesis with SuperScript (Life Technologies) and real-time PCR was performed on BioRad MyIQ system using IQ SyberGreen-Supermix from BioRad, Inc. Experiment was performed three times and samples were run in triplicate.

**Figure Legends**

Figure S1: Expression of caldesmon and calponin by western-blot is increased in cells transfected with TRIP-1 siRNA.

Figure S2: Real-time PCR analysis of TGF-β -dependent genes A) PAI-1 and B) CTGF in cells with decreased TRIP-1 expression, with or without TGFβ 48 hours treatment.

Figure S3: AKT inhibitor II treatment decreases phosphorylation of AKT. Cells in complete media were treated with vehicle (DMSO) or 40 μM AKT inhibitor for 2 hours, then samples were made and SDS PAGE gels run, and Western-Blot performed for P-AKT and Tubulin.
